# Early life experience influences dispersal in coyotes (*Canis latrans*)

**DOI:** 10.1093/beheco/arab027

**Published:** 2021-04-21

**Authors:** Emily Zepeda, Eric Payne, Ashley Wurth, Andrew Sih, Stanley Gehrt

**Affiliations:** 1 Department of Environmental Science and Policy, University of California at Davis, 1 Shields Ave., Davis, CA 95616, USA; 2 School of Environment and Natural Resources, Ohio State University, Columbus, OH, USA

**Keywords:** *Canis latrans*, dispersal, natal habitat preference induction, urban ecology

## Abstract

Natal dispersal plays an important role in connecting individual animal behavior with ecological processes at all levels of biological organization. As urban environments are rapidly increasing in extent and intensity, understanding how urbanization influences these long distance movements is critical for predicting the persistence of species and communities. There is considerable variation in the movement responses of individuals within a species, some of which is attributed to behavioral plasticity which interacts with experience to produce interindividual differences in behavior. For natal dispersers, much of this experience occurs in the natal home range. Using data collected from VHF collared coyotes (*Canis latrans*) in the Chicago Metropolitan Area we explored the relationship between early life experience with urbanization and departure, transience, and settlement behavior. Additionally, we looked at how early life experience with urbanization influenced survival to adulthood and the likelihood of experiencing a vehicle related mortality. We found that coyotes with more developed habitat in their natal home range were more likely to disperse and tended to disperse farther than individuals with more natural habitat in their natal home range. Interestingly, our analysis produced mixed results for the relationship between natal habitat and habitat selection during settlement. Finally, we found no evidence that early life experience with urbanization influenced survival to adulthood or the likelihood of experiencing vehicular mortality. Our study provides evidence that early life exposure influences dispersal behavior; however, it remains unclear how these differences ultimately affect fitness.

## Teaser

Early life experience with urbanization influences dispersal tendency and dispersal distance. Coyotes living in a large metropolitan area were less likely to disperse and traveled shorter distances during dispersal if they had more natural habitat in their natal home range. Given that dispersal is a costly behavior, coyotes born in nature preserves may be at a reproductive advantage compared to their urban counterparts.

## INTRODUCTION

Natal dispersal makes up the bulk of most species’ long distance movements (Studds et al. 2008). These movements influence ecological processes at multiple levels of biological organization. Natal dispersal affects individual fitness (Clobert et al. 2012), population extinctions and colonizations (Bowler and Benton 2009), gene flow for dispersers, and the codispersers that move with them (Trakhtenbrot 2005; Cowie and Holland 2006), and species invasions (Shigesada and Kawasaki 2002).

Natal dispersal consists of three stages: departure from the natal home range, transience through the matrix environment, and settlement in the new home range (Ronce 2007). For many species, dispersal is a plastic, condition dependent behavior. Due to the high cost of dispersal, the development of an adaptive dispersal strategy is essential to the survival and fitness of the animal (Bonte et al. 2012). For behaviorally flexible natal dispersers, the natal habitat is thought to play a significant role in shaping this behavior (Davis 2008).

The natal habitat influences departure in myriad ways. Most obvious is the relationship between departure and the quality of the natal habitat: departure rates tend to increase as habitat quality decreases (Lurz et al. 1997; Lin and Batzli 2001; Baguette et al. 2011; Legrand et al. 2015; but see Baines and McCauley 2018). Changing environmental conditions, including resource availability, competition, and predation rates, require that the animal assesses the relative qualities of the natal and matrix habitats to make an adaptive decision about whether it should stay or go (Schtickzelle and Baguette 2003; McCauley and Rowe 2010). The natal habitat can also influence departure by influencing how the animal assesses habitat quality (Stamps et al. 2009). Natal habitat preference induction (NHPI) describes the process in which an animal develops a preference for habitat features experienced in the natal home range (Davis and Stamps 2004). Although it is usually studied in the context of settlement, this induced habitat preference is likely to influence each stage of the dispersal process. For instance, NHPI may discourage an animal from departing the natal home range if it does not perceive the matrix environment as suitable habitat, whether or not it is (Benard and McCauley 2008; Piper et al. 2013).

Transience is a particularly risky stage of dispersal. During transience, the animal moves through unfamiliar matrix habitat where it is vulnerable to predation, depletion, and injury (Bonte et al. 2012). Similarly to departure, NHPI can influence how an animal perceives various habitats and environmental features during transience, where it decides to go, and how long it will search for suitable habitat. This is critical because transience length is positively associated with mortality rate (Johnson et al. 2009; Cox and Kesler 2012). Importantly, early life experiences in the natal habitat can act as a primer, influencing an animal’s ability to navigate the challenges of the matrix environment (Clobert et al. 2009; Sih 2011; Frankenhuis and Del Giudice 2012). For example, coral reef damselfish (*Pomacentrus wardi*) who were exposed to predator cues as fry had higher survival rates as adults than fish with no early life predator experience (Lönnstedt et al. 2012).

Finally, NHPI has been shown to have an effect on settlement behavior across taxa (Selonen et al. 2007; Mabry and Stamps 2008; Dixson et al. 2014; Camacho et al. 2016; Sanz-Pérez et al. 2018). Species that experience NHPI tend to select habitats similar to those found in their natal home range, consequently they are more likely to settle in that type of habitat. This can be adaptive when it allows individuals to more easily identify suitable habitat in a heterogeneous landscape. Additionally, early experience with certain habitat features can result in the development of phenotypes that are best suited for those habitats (Stamps and Davis 2006). Therefore, NHPI can confer an adaptive advantage on animals who choose habitats for which their phenotype is best suited.

Like other types of behavioral plasticity, the plastic dispersal behavior discussed above should be particularly adaptive in heterogeneous environments (Snell-Rood 2013). This is the case in many urban areas, where habitat fragments are interspersed between developed areas of different intensities. Increases in human activity and the rapid loss and fragmentation of habitat resulting from urbanization can have profound effects on animal movement, including dispersal (Ricketts 2001; van der Ree et al. 2015; Tucker et al. 2018). While dispersal is costly no matter the environment, increased detection by humans and collisions with vehicles can make urban areas particularly dangerous for dispersers (Baker and Harris 2007). However, not all individuals’ or species’ dispersal behavior is negatively impacted by urbanization. Fey et al. (2016) demonstrated that during dispersal, red squirrels (*Sciurus vulgaris*) cross roads with high traffic volume with little risk of mortality.

Coyotes are an ideal animal for studying dispersal behavior and NHPI in urban environments. They exhibit high levels of behavioral plasticity and are one of the few large carnivores to establish populations in almost every major city in North America (Poessel et al. 2017). In urban areas, they decrease risks associated with travelling through the environment by avoiding humans spatially and temporally (Murray and St. Clair 2015; Ellington and Gehrt 2019). In addition to their behavioral flexibility, coyotes are ideal for studying the influence of natal experience on dispersal because of their strong tendency to disperse. A study conducted by Harrison (1992) found that of the coyotes collared, 80% dispersed within their first year of life. In natural systems, departure from the natal home range often occurs in response to social pressure from parents which is influenced by aspects of habitat quality (Bekoff and Wells 1986, Gese et al. 1996). Finally, earlier research suggests coyotes may experience NHPI. Studies by Sacks et al. (2004; 2008) revealed that habitat type is a strong predictor of the genetic structure of the population of coyotes in central California which the authors suggest is a result of natal habitat biased dispersal.

Given the rapid rate of urbanization and the critical role dispersal plays in individual, population, and community processes, understanding how urbanization impacts dispersal behavior is important in predicting species and community responses. Despite the potential importance of NHPI in shaping adaptive responses to urbanization, to our knowledge there are few studies that explore the phenomenon in these environments and even fewer that study the behavioral pattern in carnivores (but see Mannan et al. 2007 and Milleret et al. 2019).

To understand the effects of natal habitat on dispersal in urban environments, we studied the departure, transience, and settlement behavior of coyotes in the Chicago metropolitan area. The heterogeneous landscape of the area is made of diverse land use types, including nature preserves and high density urban development. We predicted that: 1) high habitat quality in natural areas would result in lower departure rates from these areas; 2) due to NHPI, coyotes who did disperse from natural areas would travel farther during transience in pursuit of natural habitat; 3) during settlement, coyotes would select habitats similar to those experienced in their natal home range; and 4) due to lack of early life experience with humans and vehicles, coyotes dispersing from natural areas would be less likely to survive to adulthood and suffer from higher rates of vehicle related mortality. To address these predictions, we looked at the relationship between proportion of developed habitat in the natal home range and the likelihood an animal would leave its natal home range, how far it traveled during transience and where it settled. We also evaluated the relationship between proportion developed habitat in the natal home range, survival to adulthood, and vehicle-related mortality to address the hypothesis that early-life experience with developed habitat better prepares coyotes to navigate this habitat.

## METHODS

**Figure 1:**
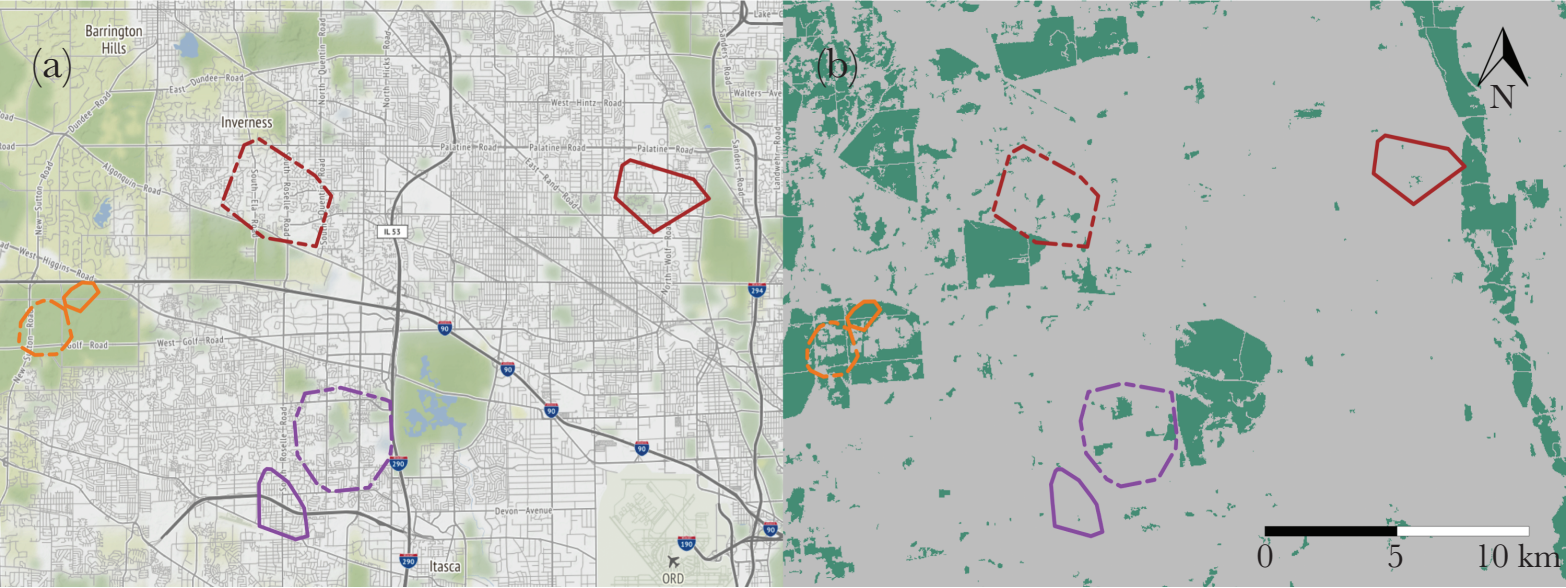
Maps showing the natal (dotted line) and adult (solid line) 95% MCP home ranges of three dispersed coyotes. Each focal coyote is represented with a different color. The hybrid map (a) is a combination of street maps and satellite maps and depicts the various levels of development coyotes might traverse during dispersal. The raster map (b) was generated using the NLCD landcover classifications and depicts “natural” (green) and “developed” (gray) habitat. This raster was used to determine the proportion of developed habitat in the natal home range, the adult home range, and in available habitat.

Coyotes included in this analysis were part of a long term study exploring the behavioral ecology, disease ecology, and management of urban coyotes (Gehrt et al. 2009; Newsome et al. 2015; Worsley-Tonks et al. 2020). Each coyote included in the study met two requirements: their parents were collared with very high frequency (VHF) transmitters in the year the coyote was born and they were VHF collared after leaving the natal den. Because juvenile movement tends to be restricted to the den site and rendezvous sites (areas frequented by members of a pack after pups have left the den) within the parents’ home range in the first few months of life (Harrison et al. 1991), we estimated the natal home range using the parents’ location data and the post-departure home range (hereinafter referred to as the “adult home range”) using the offspring’s location data (see Figure 1 for examples). Using these home ranges, we quantified characteristics of each stage of the dispersal process. This included departure from the natal home range, transience distance, and habitat type pre- and post-dispersal. Using data collected postmortem, we explored how natal habitat type influenced the likelihood a coyote survived to adulthood and the likelihood a coyote experienced a vehicle related mortality.

### Study area

The Chicago metropolitan area includes Chicago and its surrounding suburbs which make up one of the largest urban centers in North America. Consequently, the region is made up of mostly developed land uses including commercial, residential and industrial areas. Notably, the Forest Preserve District of Cook County maintains protected areas which amount to 70,000 acres or 11% of land cover in the county (Wang and Moskovits 2001). The preserves are patchily distributed throughout the landscape providing habitat for wild flora and fauna.

We characterized the landscape into habitat types using the 2016 National Land Cover Database (United States Geological Survey). This database classifies the landscape into 16 land cover categories at a 30 m resolution. We reclassified these categories into two groupings, “natural” and “developed.” Natural habitat included forest, shrubland, grassland, wetlands, and water. Developed habitat includes areas with more than 20% impervious surface cover. We included cultivated land in developed habitat due to its relatively high level of human disturbance.

### Live captures and telemetry

Coyotes used in this study were captured year round between 2000 and 2018. Trapping was done opportunistically in nature preserves and on private properties across the Chicago metropolitan area using foot-hold traps or cable restraints. After animals were captured, they were transported to a laboratory where they were immobilized with Telazol (Zoetis Manufacturing & Research) and fitted with VHF radiocollars (Advanced Telemetry Systems and Lotek Wireless). Each coyote was weighed and sexed. Blood samples were collected and later used to determine parent–offspring relationships. All procedures were approved by Ohio State University’s Institutional Animal Care and Use Committee (Protocol Nos. 2006A0245, 2010A00000113, 2013A00000012).

Coyotes were located using triangulation with a truck mounted antenna or by visual observations. Triangulations were recorded using a minimum of three bearings with a maximum of twenty minutes between first and final bearings. Coordinates were recorded with the program LOCATE II (Pacer). Coyotes were located once during the day, typically two or three times per week, and at night during tracking shifts in which we focused on a group of coyotes and obtained sequential locations at 60–120 minute intervals for 5–6 hours during the night. When radiocollared coyotes could not be located by vehicle, we conducted flights with a helicopter or fixed wing aircraft to locate signals and then confirmed their location on the ground. Such flights were deployed opportunistically in most years, and covered northeastern Illinois and parts of Wisconsin and Indiana.

### Determining parent–offspring relationships

To confirm that the correct parent location data were being used to generate each focal coyote’s natal home range, parent–offspring relationships were established using blood or tissue samples collected at the time of capture. Individuals were genotyped using 12 microsatellite markers and scored using Genetic Analysis System Software (version 8.0, Beckman-Coulter, Inc., Fullerton, California). Genotypes of pups were matched with parents using the programs CERVUS and PASOS (version 1.0; Duchesne et al. 2005). Further details can be found in Hennessy et al. (2012).

### Calculation and analysis of home range, dispersal, and mortality characteristics

#### Departure

The departure analysis examined the relationship between natal home range habitat type and the likelihood of departing from the natal home range. Eighty-five offspring from 44 parent coyotes were used in this analysis. Coyotes were categorized as dispersers if their adult home range had no overlap with their natal home range. Natal home ranges were calculated using the location data of parents from the year the focal coyote was born (number of locations used to calculate natal home ranges: mean = 306 ± 231). We only included offspring whose parents had at least 60 location observations recorded in the year the offspring was born. We calculated 95% minimum convex polygons (MCP) for each focal coyote’s parent in the package adehabitatHR (*v 0.4.18*; Calenge 2006) in R 4.0.2 (R Core Team 2020). Due to the high degree of overlap between the space use of mated pairs, when location data were available for both parents, we combined those data and calculated one MCP (Chamberlain et al. 2000). The focal coyote’s adult home ranges were calculated in the same way using the last six months or less of its location data (number of locations used to calculate adult home ranges: mean = 60 ± 31; duration of tracking for adult home ranges in days: mean = 160 ± 42). Subsetting the offspring location data was necessary because some of the individuals were collared prior to departure resulting in MCPs that spanned natal and adult home ranges. Previous studies indicate that coyote dispersal begins when coyotes are 6 months old and that most coyotes disperse within their first year of life (Harrison 1992). By focusing on the last 6 months of data, we ensured that all of the coyotes experienced this typical dispersal window, in other words, all coyotes were at least 1 year old by the end of the 6 month tracking period. Due to the challenges associated with collecting dispersal data, we included all offspring regardless of the number of recorded locations.

The proportions of developed habitat in the natal home range and the adult home range were calculated using the *raster* package in R and the NLCD raster (*v3.0–12*; Hijmans 2015). We evaluated the relationship between departure and proportion of developed habitat in the natal home range by constructing a generalized linear model (GLM) with the binomial outcome variable, dispersed or not dispersed. Proportion developed habitat in natal home range and sex were included as fixed effects.

#### Transience distance

The transience analysis assessed the relationship between natal home range habitat type and dispersal distance. First, we had to determine which coyotes were residents who had completed dispersal and which coyotes were transients who had yet to establish a home range. To determine whether a dispersed coyote was a resident, we calculated the area of the minimum convex polygon for all of the offspring using successively greater proportions of their location data, starting with the location data collected in their first 2 weeks of tracking and increasing the time frame by 2 weeks until their entire tracking period was included. We looked for individuals whose home range size reached an asymptote (Supplementary Appendix 1). We interpreted the asymptote as an indication that an animal was using the same areas throughout its tracking period and was a resident animal. Once animals were determined to be residents, their transience distances were calculated by measuring the distance between the centroid of the natal and adult home ranges using the package *rgeos* in R (*v0.5.5*, Bivand et al. 2017).

To test the relationship between dispersal distance and proportion of developed habitat in the natal home range, we constructed a GLM using a gamma distribution for the outcome variable, dispersal distance. Proportion developed habitat in natal home range, proportion developed habitat in available habitat, and sex were included as fixed effects.

We used three alternative methods to identify the area (and thus proportion of developed habitat in that area) available to coyotes during dispersal and settlement (Supplementary Appendix 2). The first, the dispersal habitat method, is best suited for coyotes with sufficient location data during dispersal (i.e., post-departure and pre-settlement). This method allowed us to evaluate the actual habitat experienced by the coyote during dispersal. For the dispersal method, we combined the location data of offspring (this included data prior to the 6 month subset period) and the location data of their parents from the year the focal coyote was born. We calculated the 100% MCPs using this combined dataset and then removed the natal home range since this area was not available to the coyotes for dispersal (our definition of dispersal excludes the natal home range). This method, however, is not informative in cases where a coyote had no or few location data collected during this time. The individualized dispersal distance method was ideal for individuals who dispersed intermediate distances because it incorporates the habitat along the direct path from the focal coyote’s natal home range to their adult home range, while also including areas where the coyote may have made exploratory bouts within a radius defined by its actual dispersal distance. For the individualized dispersal distance method, we drew a circle of available habitat around the centroid of the natal home range with a radius equal to the dispersal distance of the focal coyote. However, particularly for individuals that settled long distances from their natal home range, the individualized dispersal distance method likely included areas that the animal did not actually experience before settling. Finally, the median dispersal distance method was useful for coyotes who dispersed short distances because it included more habitat than the dispersal habitat and individualized dispersal distance methods, accounting for exploratory bouts the coyotes likely made. For the median dispersal distance method, we drew a circle of available habitat around the centroid of the natal home range with a radius equal to the median dispersal distance of all dispersal distances in the sample. While each of these methods has weaknesses, our confidence in results is enhanced if the different methods produce the same qualitative results. We ran three versions of the dispersal distance model, one for each of the three different habitat availability metrics.

#### Settlement: natal habitat preference induction

To determine if coyotes from the Chicago metropolitan area experience NHPI, we examined the relationship between the proportion of developed habitat in the natal home range, the adult home range, and in available habitats that the individual coyote could have potentially settled in. The latter is important for testing whether the habitat type in the offspring’s adult home range resembles its natal home range more than we would expect by random chance. This analysis included 19 resident dispersers.

We examined the relationship between proportion of developed habitat in the natal and adult home ranges by constructing a linear model using the Manly-Chesson index α as the outcome variable (Chesson 1978; Minder and Pyron 2018). The Manly-Chesson index α is calculated as follows:

$$\begin{array}{l} \alpha =\frac{r_{i}/p_{i}}{\sum r_{i}/p_{i}},i=1,...,m \end{array}$$

Where *r*_i_ = the proportion of used habitat type i, *p*_i_ = the proportion of available habitat type i, and *m* = the number of habitat types. Here, we simplified habitat types into two types: developed versus natural, that is, *m* = 2. We calculated the Manly-Chesson index α for developed habitat. If α = 0.5 then habitat is used randomly; there is no preference. If α > 0.5 developed habitat is selected for and if α < 0.5, developed habitat is avoided. We included the proportion of developed habitat in natal home range and sex as fixed effects in the model. We ran three separate NHPI models, one for each of the different availability metrics. NHPI is supported if there is a positive relationship between developed habitat in the individual’s natal home range and α, its preference for developed habitat.

#### Mortality

Of the 85 coyotes in the original dispersal analysis, 48 were recovered postmortem. Mortality data for these coyotes included approximate date of death and the suspected cause of death. In particular, we were able to identify mortality due to vehicle collisions with high confidence. With these 48 coyotes, we conducted two mortality analyses. The first assessed the relationship between survival to adulthood, that is, 2 years, and developed habitat in the natal home range. We constructed a Cox proportional hazards regression model, including the proportion of developed habitat in the natal and adult home ranges and sex as fixed effects. The second analysis assessed the relationship between mortality due to vehicle collision and developed habitat in the natal home range. We ran a GLM with the binomial outcome variable, death by vehicle or by other cause. Proportion developed habitat in natal and adult home ranges were included as fixed effects.

#### Statistical analysis

Statistical analyses using linear models were performed using the *stats* package (*v 3.6.2;*R Core Team 2020). We report parameter estimates, standard errors, *t*-values, and *P-*values for parameters in these models. GLMs were formatted with the package glmmTMB (*v 0.2.3*; Brooks et al. 2017). The Cox proportional hazards regression model was performed using the *survival* package (Therneau 2020). We report parameter estimates, standard errors, *z*-values, and *P*-values for parameters in these models.

## RESULTS

### Departure

**Figure 2:**
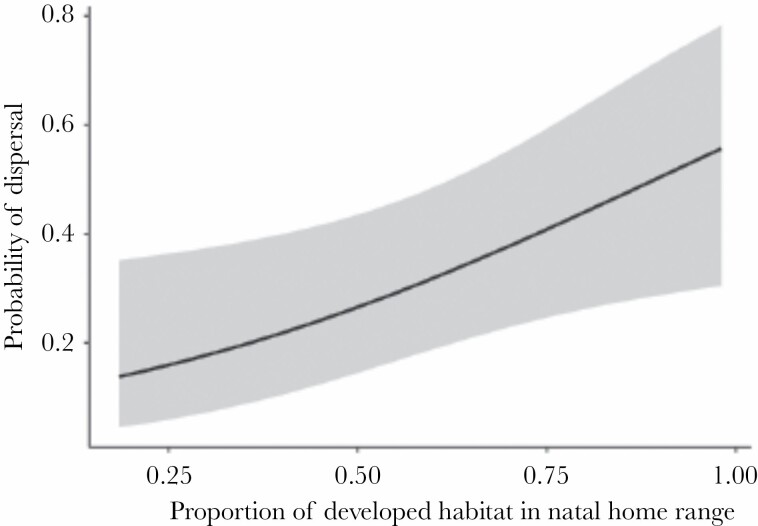
Model predicted effect of the proportion of developed habitat in the natal home range on departure. The model included sex as a fixed effect. Shaded region is the 95% confidence interval.

To assess the influence of experience in the natal home range on the propensity of coyotes to disperse, we analyzed the relationship between the proportion of developed habitat in the natal home range and departure. Of the 85 coyotes included in the analysis, 22 had no overlap between their adult and natal home ranges, satisfying our criteria for dispersal (see Table 1 for the dispersal status of all coyotes). Of those 22 coyotes, 14 had natal home ranges that consisted of more than 50% developed habitat. According to our model, proportion of developed habitat in the natal home range was positively associated with dispersal tendency (estimate = 2.591 ± 1.130, *z* = 2.292, *P* = 0.022). Coyotes with the largest proportion of developed habitat in their natal home range (0.97) were 2.7 times more likely to disperse than coyotes with the smallest proportion of developed habitat in their natal home range (0.22; Figure 2). Sex was not a significant predictor of dispersal (estimate = −0.705 ± 0.522, *z* = −1.350, *P* = 0.177).

**Table 1:** The dispersal status of the 85 coyotes included in the departure analysis. Coyotes categorized as “successfully dispersed” were those who exhibited no natal home range overlap. Animals categorized as “dispersal incomplete” exhibited natal home range overlap but were recovered postmortem outside of their natal home range. Animals who were categorized as “did not disperse” were animals who exhibited overlap with their natal home range and who died in the natal home range. Finally, animals with an “unknown” dispersal status were those who exhibited natal home range overlap and who were not recovered postmortem

Dispersal Status	Number of coyotes
Successfully dispersed	22
Dispersal incomplete	24
Did not disperse	12
Unknown	27

### Transience

**Figure 3:**
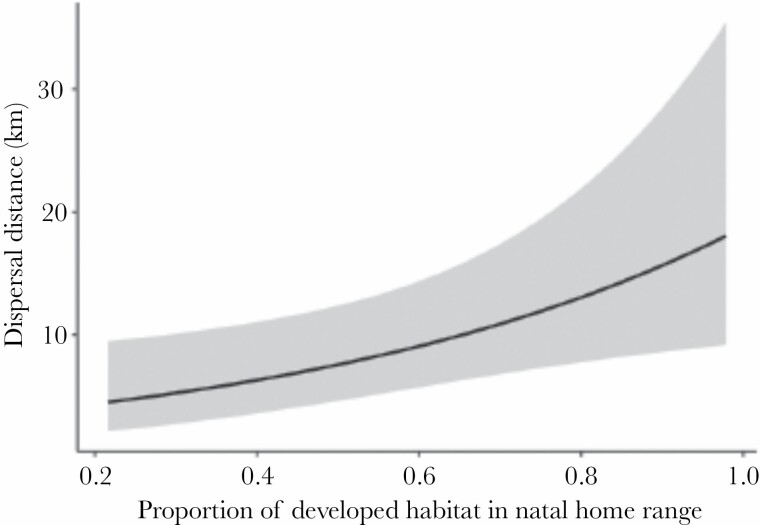
Model predicted effect of proportion of developed habitat in the natal home range on dispersal distance. The model included proportion of developed habitat in available habitat as a fixed effect. Predictions depicted here were generated using the dispersal habitat method for determining available habitat. Shaded region is the 95% confidence interval.

We analyzed the influence of experience in the natal home range on the transience behavior of coyotes by examining the relationship between the proportion of developed habitat in the natal home range and dispersal distance. Dispersal distances in this study ranged from 1.7 to 60.0 km (mean = 18.1 ± 3.7 km; median = 8.1 km). The proportion of developed habitat in the natal home range had a significant and positive relationship with dispersal distance in models using each of the three availability methods (Table 2). The model using the dispersal habitat method predicted that coyotes with the highest proportions of developed habitat in their natal home range would travel 3.9 times as far as coyotes with the lowest proportions of developed habitat in their natal home range (Figure 3). The proportion of developed habitat in habitat available while in transience was only a significant predictor in the model using the median dispersal distance method for determining habitat availability. Based on this model, the proportion of developed habitat in both the coyotes’ natal habitat and in available habitat during transience tended to be associated with longer dispersal distances. Sex was not a significant predictor in any of the models.

**Table 2:** Parameter estimates for the dispersal distance analysis. The model (distance ~ 1 + natalDeveloped + availableDeveloped + sex) included distance dispersed (distance; km) as the outcome variable. The proportion of developed habitat in the natal home range (natalDeveloped) and in the available habitat (availableDeveloped) and sex (sex) were included as fixed effects. The analysis was conducted three times, each with a different method for determining habitat availability

Availability metric	Predictor	Estimate	Std. error	*z* value	*P* value
* Dispersal habitat method*	(Intercept)	−0.098	0.575	−0.171	0.865
	natalDeveloped	1.819	0.659	2.761	0.006**
	availableDeveloped	1.832	0.740	2.474	0.013*
	sex (m)	−0.073	0.336	−0.219	0.827
* Individualized dispersal distance method*	(Intercept)	0.164	0.989	0.166	0.868
	natalDeveloped	2.095	0.696	3.011	0.003**
	availableDeveloped	1.065	1.190	0.895	0.371
	sex (m)	0.019	0.435	0.045	0.964
* Median dispersal distance method*	(Intercept)	1.145	2.910	0.394	0.694
	natalDeveloped	2.297	0.787	2.917	0.004**
	availableDeveloped	−0.279	3.690	−0.076	0.940
	sex (m)	−0.180	0.380	−0.474	0.636

*<0.05; **<0.01.

### Settlement

To understand if experience with the natal home range habitat influences preference for that habitat type during settlement, we analyzed the relationship between natal home range habitat type and the selection for developed habitat in the adult coyotes. Interestingly, the proportion of developed habitat in the natal home range was only a significant predictor of selection for developed habitat in one of the models (Table 3). With this model, a higher proportion of developed habitat in the natal home range was associated with a stronger preference for developed habitat in the adult home range; however, overall selection for developed habitat was weak. Individuals with the lowest levels of developed habitat in their natal home range were predicted to exhibit a strong avoidance of developed habitat (α = 0.10) while animals with an average proportion of developed habitat in their natal home range still slightly avoided developed habitat (α = 0.44). Animals with the highest levels of developed habitat in the natal home range were still predicted to exhibit only a weak preference for developed habitat (α = 0.58). Sex was not a significant predictor in any of the models.

**Table 3:** Parameter estimates for the natal habitat preference induction analysis. The model (selectionDeveloped ~ 1 + natalDeveloped + sex) included the Manly-Chesson index α (selectionDeveloped) as the outcome variable. The proportion of developed habitat in the natal home range (natalDeveloped) and sex (sex) were included as fixed effects. The analysis was conducted three times, each with a different method for determining habitat availability

Availability metric	Predictor	Estimate	Std. error	*t* value	*P* value
*Dispersal habitat method*	(Intercept)	0.365	0.174	2.104	0.052
	natalDeveloped	0.219	0.223	0.985	0.340
	sex (m)	0.012	0.117	0.106	0.917
*Individualized dispersal distance method*	(Intercept)	0.045	0.247	0.181	0.859
	natalDeveloped	0.578	0.317	1.825	0.087
	sex (m)	0.147	0.166	0.886	0.389
*Median dispersal distance method*	(Intercept)	−0.021	0.227	−0.092	0.928
	natalDeveloped	0.620	0.292	2.127	0.049*
	sex (m)	0.055	0.153	0.357	0.726

*<0.05; **<0.01.

### Mortality

We analyzed the relationship between survival to adulthood and natal habitat type. Survival to adulthood (age 2) was quite high with only 11 of 48 coyotes dying before reaching adulthood; however, survival (or not) to adulthood was not explained by either the proportion of developed habitat in the natal home range (estimate = −0.779 ± 0.773, *z* = −1.007, *P* = 0.314), the proportion of developed habitat in the adult home range (estimate = 1.414 ± 0.761, *z* = 1.859, *P* = 0.063), or sex (estimate = −0.222 ± 0.272, *z* = −0.816, *P* = 0.414).

To understand if experience with developed habitat in the natal home range influenced the likelihood a coyote experienced a vehicle related death, we analyzed the relationship between natal home range habitat type and vehicle related mortality. Twenty-eight of the 48 coyotes included in the mortality analysis died after being hit by a vehicle. Neither the proportion of developed habitat in the natal home range (estimate = −0.529 ± 1.519, *z* = −0.349, *P* = 0.727) nor the proportion of developed habitat in the adult home range (estimate = −0.789 ± 1.633, *z* = −0.484, *P* = 0.629) had a significant effect on the probability of a coyote experiencing a vehicle related death.

## DISCUSSION

We used VHF data from a long term study of urban coyotes to explore the relationship between early life experience with urbanization and dispersal and mortality. We found evidence that coyotes from natal home ranges with more developed habitat were more likely to disperse than animals from primarily natural habitats. Contrary to our prediction, of the dispersing coyotes, coyotes with more developed habitat in their natal home range dispersed farther. Models using different habitat availability metrics to explore the relationship between natal habitat type and adult habitat selection produced mixed results; however, we found some evidence that coyotes experience NHPI. Finally, we found no evidence that experience with developed habitat in the natal home range influences survival to adulthood or the likelihood of experiencing a vehicle-related mortality.

Early life experience may shape juvenile coyotes’ perceptions of habitat quality and their willingness to depart from the natal home range. While urban wildlife generally avoid humans and their associated landscapes, there is evidence that avoidance is plastic and varies with experience (Kitchen et al. 2011; Vincze et al. 2016). Studies comparing the behavioral traits of urban and rural coyotes found that urban coyotes tend to be bolder and more exploratory toward humans and novel objects (Breck et al. 2019; Brooks et al. 2020). Additionally, Schell et al. (2018) identified a flexible, transgenerational mechanism for human tolerance in coyotes where successive litters born to the same mated pair exhibited increased habituation towards humans as the parents' became more habituated . In addition to responses to individual environmental features, larger scale changes in preference for human altered habitats have been observed in other mammalian species (Knopff et al. 2014; Scrafford et al. 2017). Raccoons in more highly urbanized areas show increased selection for human-use areas, which may be a response to their experience using anthropogenic resources in these areas (Bozek et al. 2007). Because the decision to leave the natal home range is dependent on perceptions of habitat quality, experience with more or less urbanization in the natal home range is likely influencing what the animal perceives as the optimal decision (Bowler and Benton 2009). Animals who primarily experience natural habitat in their early life might perceive the surrounding matrix as more hostile decreasing their motivation to initiate dispersal relative to those with more experience in urban habitats.

In addition to environmental factors, social interactions play an important role in dispersal (Wey et al. 2015). For coyotes, intrapack interactions are particularly important in determining departure behavior (Bekoff 1977). Mated pairs will often engage in antagonistic interactions with yearlings or older offspring during the mating season, driving them out of the natal home range. However, increased resource availability in natural areas may reduce social pressure to disperse. Interestingly, anecdotal evidence for alternative “dispersal” behavior has been observed in this system. In nature preserves, some coyotes have been observed to take over part of their natal home range causing shifts in their parents’ home range away from that area (Gehrt, unpublished data). This behavior is likely less frequent or absent in developed habitat particularly if lower resource availability results in increased competition and social pressure to disperse (Gese et al. 1996).

Transience is risky (Bowler and Benton 2009). In undisturbed areas, dispersers are more likely to die during transience than their nondispersing counterparts and this increases with distance travelled during transience (Bekoff and Wells 1986; Bonnet et al. 1999; Letty et al. 2000; Meek et al. 2003; Moehrenschlager and McDonald 2003). In urban areas, risk may be enhanced as animals are required to navigate through unfamiliar matrix habitat where various human and vehicle related dangers are common. Dispersal distances in this study (mean: 18.1 ± 3.7 km) were substantially shorter than mean distances observed in less disturbed areas, which range from 51 to 310 km (Harrison 1992; Kolbe and Squires 2004; Sasmal et al. 2019). In our study, data collection methods bias the sample toward individuals who disperse within the area of high tracking effort. However, other studies indicating that habitat fragmentation inhibits animals’ movement suggest that the patchy, developed landscape of the Chicago metropolitan area may also contribute to shorter dispersal distances (Tucker et al. 2018). Shorter dispersal distances in response to human activity or development could reduce gene flow and thus facilitate evolutionary adaptation to urbanization. In particular, if human altered habitats act as a barrier to wildlife dispersing from more remote habitats in the same way that they inhibit the dispersal movements of animals in our study, urban populations may undergo microevolution at a more rapid rate than would be expected if immigration rates remained at undisturbed levels (Sol et al. 2013; Miranda 2017; Adducci et al. 2020). Although for a behaviorally flexible generalist like the coyote, phenotypic plasticity is generally considered the most salient response to human disturbance, in many taxa, genetic adaptations are also important (Atwell et al. 2012; Miranda et al. 2013; Mueller et al. 2013; Alberti et al. 2020; Lambert et al. 2020).

We found that of the coyotes who did disperse, those with more urban development in their natal home range tended to disperse farther. Larger home ranges in developed habitat may force coyotes from these areas to disperse farther to find suitable, unoccupied habitat (Gehrt et al. 2009). While this may put developed coyotes at a disadvantage due to the risks they might face travelling long distances through the urban matrix, given coyotes’ high degree of behavioral plasticity and their early life experience with developed habitat we hypothesized that these coyotes are better at navigating these risks. Studies examining the relationship between behavioral responses to human disturbance and previous experience have found that experience with humans and human altered habitats plays an important role in shaping adaptive behavior (Zaccaroni et al. 2007; Thurfjell et al. 2017). Despite this evidence, our mortality analyses did not indicate that this phenomenon occurs in these coyotes. This might be attributed to our limited sample size. However, it is possible that early life experience with anthropogenic risks and increased exposure to anthropogenic risks during dispersal have additive effects in developed coyotes resulting in no difference in the likelihood that they and their natural counterparts will survive to adulthood or experience a vehicle related mortality.

While previous studies have suggested coyotes may experience NHPI (Sacks et al. 2004, Sacks et al. 2008), the mixed results in our study are open to multiple interpretations. If the median dispersal distance method for establishing availability is most accurate, coyotes may exhibit NHPI. In that case, habitat preferences formed in the natal home range could have important population-level effects. For instance, if only coyotes with the highest proportion of developed habitat are settling in highly urbanized areas and coyotes born in more natural versus more developed habitat within the Chicago metropolitan area are dispersing to and mating with individuals from the same habitats, this assortative mating could change the scale at which microevolution might act as an adaptive mechanism (Richardson et al. 2014). Instead of interbreeding between all coyotes in the region, genetically distinct subpopulations within highly urbanized areas may be undergoing selection specific to that habitat. In addition to its effects on individual fitness and population dynamics, it could also have implications for human–coyote interactions. Many of the behavioral traits that allow animals to take advantage of novel opportunities and cope with novel challenges in urban environments are also traits that increase the likelihood an animal interacts with humans (Barrett et al. 2019). Studies comparing traits including neophobilia and boldness in urban and rural passerines found evidence that some of the differences in behavior between populations can be attributed to microevolution (Atwell et al. 2012; Miranda et al. 2013; Müller et al. 2013). Associative breeding among the least neophobic and most bold coyotes has the potential to enhance human-coyote conflict in these areas.

Despite some evidence for NHPI, the lack of a significant relationship between natal habitat type and selection during settlement in our other two model suggests that in these coyotes, the phenomenon may not be occurring or may at best, be weak. There are a number of explanations for an absence of NHPI. Coyotes may have an innate preference for natural areas that outweighs early life experiences. Again, this is supported by previous research that indicates coyotes prefer natural or rural areas to urban areas (Tigas et al. 2002; Gehrt et al. 2009; Gese et al. 2012; Poessel et al. 2016; Wang et al. 2017, Ellington and Gehrt 2019). Alternatively, given the high costs of searching for and establishing a new home range, other intrinsic characteristics like condition or extrinsic factors like competition may drive settlement decisions rather than previous experience with a given habitat type (Clobert et al. 2009; Rémy et al. 2014; Wey et al. 2015). Finally, for animals that exhibit high levels of plasticity, if early life experience is not reinforced, its effects on behavior may be updated to reflect more recent experiences. This has been shown in rats (*Rattus spp.)* whose behavioral reactivity to stress, which increases after experiencing early life maternal separation, can be reversed with environmental enrichment (Francis et al. 2002). The results of these two models agree with a number of studies that found no evidence of NHPI suggesting innate preferences or experiences other than the natal habitat may be more important for shaping dispersal behavior in some species (Reiskind and Zarrabi 2013; Ousterhout 2014).

## CONCLUSION

Despite dispersal’s importance at various levels of biological organization, few wildlife studies have examined the stages of dispersal beyond departure and even fewer have looked at this behavior in an urban setting. We show that early life experience with urbanization influences departure, transience, and potentially settlement behavior. While we did not find that these differences resulted in changes to survival to adulthood, we suspect that they may have implications for individual fitness, population structure, and human–wildlife interactions.

Future studies should focus on fine scale heterogeneity in intrinsic and extrinsic factors associated with dispersal. Intrinsic traits like condition and behavioral type are important factors that interact with the environment to produce variation in dispersal behavior. Additionally, more detailed information about the natal and matrix environments should be considered. While we were constrained to two habitat types based on our sample size, considering multiple dimensions of environmental variability like vegetation density and type, level of impervious surface cover, patch size, human population density, road density, and traffic rate will allow the identification of specific urban environmental factors that drive differences in dispersal behavior. These are lost when the landscape is dichotomized into categories like developed and natural or urban and rural. Combining detailed features about the animal and its environment may help identify how they interact to produce dispersal behavior. Finally, gaining a deeper understanding of how various dispersal strategies are associated with survival and fitness will be crucial in identifying which dispersal responses are truly adaptive and how those strategies influences dynamics at the various levels of biological organization.

## FUNDING

This work was supported by Cook County Animal and Rabies Control, the Max McGraw Wildlife Foundation, Forest Preserve District of Cook County, and the National Science Foundation Graduate Research Fellowship (grant number 1650042 to E.Z.).

This longer research study has benefited immensely from the hard work and dedication of many field technicians, graduate students, and project personnel. We thank Chris Anchor for his support of this work. We are particularly indebted to Shane McKenzie for his leadership and organizational skills on this project.

## Data availability

Analyses reported in this article can be reproduced using the data provided by Zepeda et al. (2021).

## Supplementary Material

arab027_suppl_Supplementary_Appendices
