# Supplementary material for: Early life experience influences dispersal in coyotes (Canis latrans)
Source: Behav Ecol. 2021 Apr 21;32(4):728–37. doi: 10.1093/beheco/arab027 (PMC8374878; doi:10.1093/beheco/arab027)
Supplement: arab027_suppl_Supplementary_Appendices [file arab027_suppl_supplementary_appendices.docx]

**Appendices for**

**“Variation in plastic consumption: social group size and membership**

**influences individual susceptibility to an evolutionary trap”**

**Appendix 1**

***Determining resident dispersers***

***
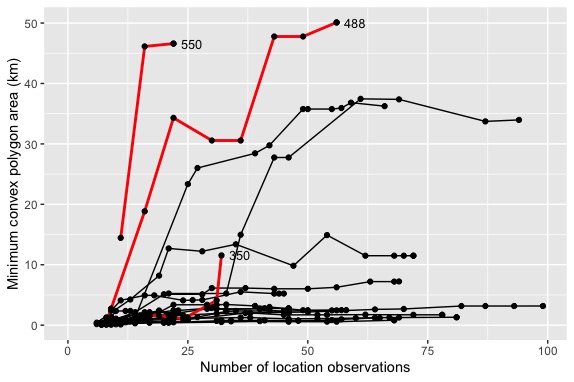
***

To determine whether a dispersed coyote was a resident we calculated the area of the minimum convex polygon for all of the focal coyotes using successively greater proportions of their location data, starting with the location data collected in their first two weeks of tracking and increasing the time frame by two weeks until their entire tracking period was included. Individuals (coyotes 550, 488 and 350; highlighted in red) whose minimum convex polygon areas did not reach an asymptote were considered transients and removed from further analyses.

**Appendix 2**

***Calculating available habitat***

Natal and adult home ranges and three habitat availability metrics. The natal home range, generated using parents’ (gray coyote) location data from the year the focal coyote (black coyote) was born, is represented in dark gray (a). The focal coyote’s home range, generated using locations collected in the last 6 months of its tracking period, is represented in black and its dispersal distance represented as the black dashed line (a). The dispersal habitat method (b) shows the minimum convex polygon used as the region of available habitat in light gray. This polygon was generated using all of the focal coyote’s location data as well as the data used to generate its natal home range. The natal home range was then removed from the available habitat polygon because it was not considered habitat that the animal could disperse to. The individualized dispersal distance method (c) draws a circle of available habitat, represented in light gray, around the center of the natal home range with the radius equal to the dispersal distance of the focal coyote (dashed line). The median dispersal distance method (d) draws a circle of available habitat, represented in light gray, around the center of the natal home range with the radius equal to the median dispersal distance in our study (dotted line).
